# Current Concepts in Disorders of Sexual Development

**DOI:** 10.4274/jcrpe.v3i3.22

**Published:** 2011-09-09

**Authors:** Gönül Öçal

**Affiliations:** 1 Ankara University School of Medicine, Department of Pediatric Endocrinology, Ankara, Turkey; +90 312 595 64 04 gonulocal@gmail.com

**Keywords:** Sex development disorders, gonads, gender assignment

## Abstract

Disorders of sex development (DSD) with or without ambiguous genitalia require medical attention to reach a definite diagnosis. Advances in identification of molecular causes of abnormal sex, heightened awareness of ethical issues and  this necessitated a re-evaluation of nomenclature. The term DSD was proposed for congenital conditions in which chromosomal, gonadal or anatomical sex is atypical. In general, factors influencing sex determination are transcriptional regulators, whereas factors important for sex differentiation are secreted hormones and their receptors.The current intense debate on the management of patients with intersexuality and related conditions focus on four major issues: 1) aetiological diagnosis, 2) assignment of gender, 3) indication for and timing of genital surgery, 4) the disclosure of medical information to the patient and his/her parents. The psychological and social implications of gender assignment require a multidisciplinary approach and a team which includes ageneticist, neonatologist, endocrinologist, gynaecologist, psychiatrist, surgeon and a social worker. Each patient should be evaluated individually by multidisciplinary approach.

**Conflict of interest:**None declared.

## INTRODUCTION

Phenotypic sex results from the differentiation of internal ducts and external genitalia under the influence of sex-determining genes and hormones (1,2,3,4,5,6,7,8). In one of every 4500 births, the genital appearance is abnormal and it is not possible to decide at first glance  the sex of the infant. Disorders of sexual development (DSD) are a very important clinical issue with its different aspects relating to diagnosis, treatment and sex of rearing. The classification of ambiguous genitalia in patients is difficult because similar or identical phenotypes may have several aetiologies (1,2,3,4,5). 

**Physiology of Sex Development**

Sex determination is the result of a series of molecular events that direct the undifferentiated bipotential gonad to become either a testis or an ovary. The bipotential gonad develops from the urogenital ridge. By 6-7 weeks of fetal life, fetuses of both sexes have two sets of internal ducts: the Müllerian ducts and the Wolffian ducts. The external genitalia at 6-7 weeks  gestation appear female and include a genital tubercle, the genital folds, urethral folds and a urogenital opening. During the bipotential stage, many genes (WT1, SF1, LHX9, LIM1, PAX2, GATA4, EMX2, WNT4) are expressed at similarly low levels in XY and XX gonadal ridges (9,10,11,12,13). However, all are necessary for normal gonadal development in both sexes, with gene dosage and relative expression levels playing an important role in the sexually divergent fate of the gonads. The steroidogenic factor 1 (SF1), Wingless-type gene (WNT4) and Wilms tumor suppressor gene 1 (WT1) are expressed in the urogenital ridge and have a role in the formation of the gonads, kidneys and adrenal cortex. SF1 located on chromosome 9p33, is important for the biosynthesis of adrenal and gonadal steroids and for the development of the ventral nucleus of the hypothalamus. SF1 is expressed in the bipotential gonad of both sexes. Expression of SF1 continues in the developing testis, but is repressed in the ovary. The WT1 gene is located on chromosome 11p13 and encodes a transcription factor necessary for the development of the bipotential gonad and the kidneys. Wilms tumor suppressor gene activates transcription of SRY (15,16). The important event in gonadal differentiation is the commitment of the bipotential gonad to become either an ovary or a testis. The essential genes affecting this process are as follows: WT1, SF1,CBX2, SOX9, fibroblast growth factor 9 (FGF9),  prostaglandin D2 (PGD2), DAX1, WNT4, forkhead family transcription factor 2 (FOXL2), R-Spondin 1 (RSPO1) and b-catenin. While SOX9, FGF9 and PGD2 have more testis-promoting activity, DAX1, WNT4, FOXL2, RSPO1 and b-catenin are predominantly ovary-promoting genes (12). SOX9/FGF9 and WNT4/RSPO1 act as antagonistic signals in early gonadal differentiation.

**Figure 1:**
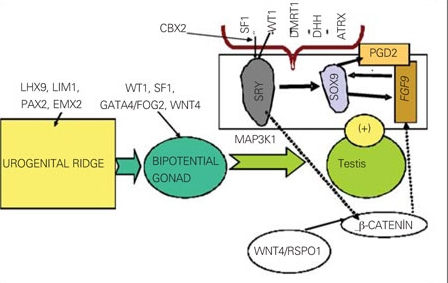
Figure 1. Factors involved in testes differentation

**Male Differentiation:**The factors involved in testicular differentiation are given in Figure 1(14,15,16,17,18,19,20,21). Male phenotypic development can be viewed as a two-step process: 1) testis formation from the primitive gonad (sexual determination) and 2) internal and external genitalia differentiation by action of hormones secreted by the fetal testis (sexual differentiation) (10). The first step  involves the interplay of several transcription factors and signaling cells (14,15,16,17,18,19,20,21).

The DAX1 gene region on Xp21 is necessary for gonadal development in both sexes.  A single copy of the DAX1 is essential for normal testicular cord formation. However, DAX1 overexpression in an XY fetus impairs testis differentiation. Testis determination occurs at about the sixth week of gestation. SRY gene that is located on the Y-chromosome (Yp11.3) initiates sex determination by downstream regulation of sex-determining factors. Expression of several genes including WT1,CBX2(M33), SF1, GATA4/FOG2 is critical to SRY activation. The SRY gene encodes a unique transcription factor that activates a testis-forming pathway (12,18,19). After expression of SRY, SOX9 (SRY-related HMG box gene) expression is upregulated in the developing testis (20). The SOX9 gene, located  on 7q24.3-25.1, is essential for early testis development.  SOX9 up-regulates PGD2 and FGF9 genes. FGF9 and PGD2 maintain SOX9 expression, forming a positive feed-forward loop in XY gonads (21). The balance between FGF9 and WNT4/RSPO1/b-catenin signals is shifted in favor of FGF9, establishing the male pathway. On the other hand, DMRT1, ATRX and DHH and several autosomal genes are also involved in testes determination (12). 

The second step in male sex differentiation is a more straightforward process. The production of anti-Müllerian hormone (AMH) by Sertoli cells and androgens by Leydig cells in a critical concentration-dependent and time-dependent manner induces male sexual differentiation by means of a hormone–dependent process. AMH acts on its receptor in the Müllerian ducts to cause their regression. Testosterone (T) acts on the androgen receptor (AR) in the Wolffian ducts to induce the formation of epidydimis, deferent ducts and seminal vesicles. The Leydig cells also produce insulin-like factor 3 (INSL3, relaxin-like factor), which causes the testes to descend to the scrotum. T is further reduced to dihydrotestosterone (DHT), which acts on the androgen receptor of the prostate and external genitalia to cause its masculinization. Binding of T or DHT to  AR is necessary for androgen effect. Various coregulators interact probably for transcriptional activity of AR. By contrast, coregulators such as corepressors repress transcriptions (22,23).

**Figure 2:**
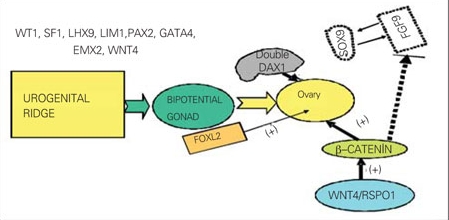
Figure 2. Factors involved in ovarian differentation

**Female Differentiation:**The factors involved in ovarian differentiation are given in Figure 2 (24,25,26,27). In the absence of SRY, the support cell precursors differentiate as granulosa cells, thus initiating the ovarian pathway. DAX1 is necessary for both testicular and ovarian development, with a need for precise gene expression dosage. Overexpression in either DAX1 or WNT4/RSPO1 antagonizes testis formation. WNT4/RSPO1/b-catenin pathway blocks FGF9 and promotes the ovarian fate (24). In XX gonads, WNT4 dominates and results in an induction of b-catenin and silencing of FGF9 and SOX9. WNT4-signaling pathway plays a major role in ovarian development and maintenance, regulation of Müllerian ducts formation and ovarian steroidogenesis (26,27,28). WNT4 has been shown to play a critical role  in the development of the reproductive system and  also in the formation of the kidneys, adrenals, pituitary gland, and mammary tissues. Absence of WNT4 leads to testis-like development within the ovary. Conversely, overexpression of WNT4 in the male leads to female sex reversal. RSPO1 is another gene essential in sex determination responsible for the protein RSPO1, which plays an important role in suppression of the SOX9 gene (27,28). Loss of function mutations in the human RSPO1 gene in mice results in the formation of ovotestes in the XX fetus (28). WNT4, RSPO1 and  b-catenin seem to have both pro-ovarian and anti-testicular activities from early embryonic life, while FOXL2  may also have similar actions postnatally.* FOXL2 is expressed early within the genital ridge of the fetal, postnatal and adult ovary and eyelids. FOXL2 is involved in granulosa cell differentiation, follicle development and maintenance during fertile life (29). Müllerian ducts give rise to the fallopian tubes, uterus and the upper two-third of the vagina. In the female, the genital tubercle becomes the clitoris, the labio-scrotal folds become the labia majora, and the urethral folds become the labia minora. 

**Nomenclature and Definitions**

Formerly, intersex disorders were subdivided into three main groups as: associated with gonadal dysgenesis, associated with undervirilization of 46,XY individuals, and conditions associated with prenatal virilization of 46,XX subjects. The nomenclature used to describe atypical sexual differentiation has since changed (2,3,4,5). Instead of using the confusing and/or controversial terms such as "intersex," "hermaphroditism" and "sex reversal", the consensus statement recommended a new taxonomy based on the umbrella term, “DSD” (3). This broad category includes common entities such as Turner syndrome and Klinefelter syndrome as well as rare disorders such as cloacal exstrophy and aphallia. Many DSDs are associated with ambiguous genitalia,  however, a few may present with delayed puberty or primary amenorrhea. The Lawson Wilkins Pediatric Endocrine Society (LWPES) and the European Society for Paediatric Endocrinology (ESPE) consensus group proposed the classification of DSDs into: 

**1) Sex chromosome DSDs**  (45,X Turner and variants, 47,XXY Klinefelter and variants, 45X/46XY mixed gonadal disgenesis (MGD) and chromosomal ovotesticular DSD “46XX/46XY chimeric type  or mosaic type”); 2) 46,XY DSDs (disorders of  testicular development or disorders in androgen synthesis/action); and 3)  46,XX DSDs (disorders of ovarian development or fetal androgen excess) (Table 1). Additional categorization based on sex chromosome complement was recommended but not clearly defined  (30,31). It is recognized that some conditions do not fit exactly into one specific diagnostic category or may be placed in more than one category (5). The DSD nomenclature has recently divided "ovotesticular DSD" (formerly true hermaphroditism) into 46,XY ovotesticular DSD, 46,XX ovotesticular DSD, and chromosomal ovotesticular DSD (46,XX/46,XY” chimerism or 45,X/46,XY” mosaic type). 

**1)Sex chromosome DSDs** (45,X Turner and variants, 47,XXY Klinefelter and variants, 45X/46XY MGD,  choromosomal ovotesticular DSD (46XX/46XY chimeric type or mosaic type): This type of DSDs is associated with a numerical sex chromosome abnormality leading to abnormal gonadal development (2,3,5,30,31,32,33,34, 35,36,37). Sex chromosome DSD was formerly termed as gonadal dysgenesis in most of the patients in this group (5). If a testis is poorly formed, it is called a dysgenetic testis, and if an ovary  is poorly formed, it is called a streak gonad. A patient with a Y chromosome is at high risk of developing a tumor in a streak or dysgenetic gonad. Klinefelter and Turner syndromes (TS) are the most frequently encountered  sex chromosomal abnormalities (34,35,36). More than half of girls with TS have chromosomal mosaicism. The most common genotype of Klinefelter syndrome is XXY, although variants exist with different numbers of X chromosome. In patients with 45X/46XY MGD, clinical manifestations are highly variable, ranging from partial virilization and ambiguous genitalia at birth to  a completely male or female phenotype. The most common feature of MGD is asymmetric development of the testes, often with a dysgenetic testis on one side and a streak gonad on the other. Asymmetrical  external and internal genitalia may also be present. The presence of 45,X cell lines is frequently associated with Y chromosome rearrangements (commonly dicentric and ring Y chromosomes), which may also have an impact on the phenotype.  Chromosomal ovotesticular DSD (chimeric type or mosaic type) is associated with ovarian and testicular tissues found in either the same or opposite gonad just as in 46,XX and 46,XY ovotesticular DSD. The genital duct develops  according to the ipsilateral gonad. 

**2) 46,XY disorders of sex development (46,XY DSD):**

 The term “male pseudohermaphrodite” was used to describe the patients with incompletely masculinized external genitalia possessing an XY chromosome.** 46,XY DSD can result either from disorders of testicular development or disorders in androgen synthesis/androgen action (6). These patients are characterized by ambiguous or female external genitalia, caused by incomplete intrauterine masculinization. Male gonad(s) are palpable in the majority of 46,XY DSD patients. Abnormalities in the expression of genes involved in the cascade of testis determination can cause anomalies of gonadal development and consequently, 46,XY DSD (complete or partial forms of gonadal dysgenesis with or without syndromic phenotype, ovotesticular DSD, testicular regression syndrome). 

Failure of testis determination results in the development of the female phenotype, while genetic alterations resulting in partial testicular development can give rise to a wide spectrum of incomplete masculinization. 46,XY partial gonadal dysgenesis,  characterized by partial testicular differentiation and  ambiguous genitalia, is usually observed in the newborn period. Mutation in WT1 gene results in Denys-Drash syndrome (without uterus) or Frasier syndrome (with uterus) characterized by 46,XY partial gonadal dysgenesis and severe renal dysfunction with or without Wilms tumor (1^6^). Complete  gonadal dysgenesis in 46,XY individuals (Swyer syndrome) is characterized by a female phenotype with full development of unambiguous female genitalia, normally developed Müllerian structures, and streak gonads. These streak gonads are removed due to their association with gonadoblastoma. In general, these patients present because of delayed puberty. In agonadism (vanishing testes syndrome, testicular regression) boys present with normal male genitalia, indicating that they  must have had testicular function in the fetal period, and bilateral anorchia. The defects in peptide hormones and their receptors as well as the timing of hormonal exposure are also critical to appropriate male development. Leydig cell aplasia/hypoplasia, due to abnormalities in hCG/LH receptor, and T biosynthesis defects (STAR deficiency, P450scc deficiency, 3-b hydroxysteroid dehydrogenase type II deficiency, 17a-hydroxylase and 17,20-lyase deficiency, isolated 17,20-lyase deficiency, P450 oxidoreductase ”POR gene” defect, 17b-hydroxysteroid dehydrogenase III deficiency) result in androgen synthesis defect. Severely affected infants for POR gene of both sexes have ambiguous genitalia. The males are undervirilized because of defective 17,20-lyase activity of P450c17. Disorders of AMH and AMH receptors result in persistent Müllerian duct syndrome (PMDS). PMDS is inherited in a sex-limited autosomal recessive manner caused by a mutation in the AMH or AMH-receptor genes. 5a-reductase type 2 deficiency (38,39,40) and complete/partial forms of androgen insensitivity syndromes (CAIS, PAIS) result in disorders of androgen action (22,23).

3) 46,XX disorders of sex development (46,XX DSD): The term female pseudohermaphrodite was used to describe the patients with 46,XX karyotype and with masculinized external genitalia. Currently, these disorders are described as 46,XX DSD.** 46,XX DSD can result either from disorders of ovarian development or fetal androgen excess (6). SRY positivity; WNT4, RSPO1,  b-catenin gene defects; and duplication of SOX9 gene lead to testis-like formation within the ovary (streak gonad, dysgenetic testis or ovotestis) in the 46,XX patients. A single copy of the WNT4 gene in females causes Müllerian abnormalities, renal abnormalies (e.g. renal agenesis), and androgen excess. Their phenotype resembles that of patients with the Mayer-Rokitansky-Küster-Hauser (MRKH) syndrome. With absence of both copies of this gene, females show male sex and SERKAL syndrome (female to male sex reversal; renal, adrenal and lung dysgenesis) (26). RSPO1 is essential in sex determination and skin differentiation. RSPO1 gene mutations lead to XX sex reversal, palmoplantar hyperkeratosis and predisposition to squamous cell carcinoma of the skin (27,28). FOXL2 mutations result in a variety of phenotypes, from adult ovarian failure to development of streak gonads (29). Mutations in FOXL2 are responsible for blepharophimosis-ptosis-epicanthus inversus syndrome (BPES) and can be associated with premature ovarian failure. Ovarian dysgenesis coexisting with sensorineural deafness is diagnosed as Perrault syndrome. In ovotesticular DSDs, the most common karyotype is 46,XX followed by 46,XX/46,XY chimerism or mosaicism, and 46,XY. Most 46,XX ovotesticular DSDs are SRY-negative, and the genes responsible have not yet been identified. A mutated downstream gene in the sex determination cascade is likely to allow for testicular determination. 46,XX testicular DSD is distinct from XX ovotesticular DSD and is associated with male habitus, small testes, azoospermia and no evidence of uterus or ovaries.

 Virilized females with two ovaries, XX karyotype and ambiguous genitalia  are usually exposed to external genitalia androgens of fetal origin or to androgens of maternal origin. The majority of virilized 46,XX infants will have congenital adrenal hyperplasia (CAH) (most commonly 21a-hydroxylase and 11b- hydroxylase or rarely 3b-hydroxysteroid dehydrogenase deficiencies). Apparent combined P450c17 and P450c21 deficiency is a rare variant of CAH.  Mutations of POR gene cause disordered steroidogenesis with prenatal virilization without worsening of postnatal virilization in female fetuses (41,42). Cytochrome POR is a protein that transfers electrons from NADPH to all microsomal cytochrome P450 enzymes and three steroidogenic enzymes, namely, P450c17 (17a-hydroxylase/17,20 lyase), P450c21 (21-hydroxylase), and P450aro (aromatase). Severely affected female infants for POR gene are virilized because of defective aromatase activity and because of the diversion of 17-hydroxyprogesterone (17OHP) to DHT via the “backdoor pathway” to androgens that bypass dihydroepiandrosterone (DHEA), androstenedione (A) and T (42). Severely affected infants also have the Antley-Bixler skeletal malformation syndrome (ABS) characterized by craniosynostosis and radio-humeral or radio-ulnar synostosis. Rarer causes of fetal androgen excess in XX infants are maternal androgen ingestion, maternal virilizing disease, fetoplacental aromatase deficiency, sulfatase deficiency,  virilizing luteoma of pregnancy, glucocorticoid receptor mutation. 

Aromatase deficiency is rare in humans (43). Aromatase is the enzyme that catalyzes conversion of androgens into estrogens, and if aromatase is nonfunctional because of an inactivating mutation, estrogen can not be synthesized. If the fetus lacks aromatase activity, DHEA produced by the fetal adrenal glands cannot be converted to estrogen by the placenta, and is converted to T peripherally. This  results in virilization of both fetus and mother. 

Cystic ovaries and delayed bone maturation can occur during childhood and adolescence in these girls. They may  present at pubertal ages with primary amenorrhea, failure of breast development except in partial cases, virilization, and hypergonadotrophic hypogonadism. 

Sulfotransferase deficiency is a monogenic cause of hyperandrogenism. DHEA sulfotransferase, known as SULT2A1, converts the androgen precursor DHEA to its inactive sulfate ester, DHEAS, thereby preventing the conversion of DHEA to an active androgen. If this pathway is blocked, more DHEA will be converted to androstenedione and hyperandrogenism may occur (44). 

**Investigation of DSD Patients** 

Optimal care of patients with DSD requires a multidisciplinary team and begins in the newborn period. A family history, prenatal history, a general physical examination with attention to any associated dysmorphic features, and an assessment of the genital anatomy are the first steps towards a correct diagnosis. The diagnostic evaluation of DSD includes hormone measurements, imaging, cytogenetic and molecular studies and in some cases endoscopic, laparoscopic and gonadal biopsy (6,7,8,45,46,50,51,52,53,54,55). The genetic evaluation includes karyotype, FISH and, more recently, specific molecular studies to screen the presence of mutations or gene dosage imbalance (AR, SRY, SF1, WT1, CYP21, SOX9, DAX-1, 17b hydroxysteroid dehydrogenase, 5a-reductase-2, and others). However, current molecular diagnosis is limited by cost, accessibility, and quality control. Ultrasonography shows the presence or absence of Müllerian/Wolfian structures and can locate the gonads and their  echo texture. Ultrasonography also can identify associated malformations such as renal abnormalities. 

**Figures 3a:**
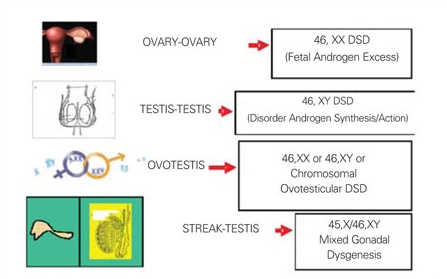
Figure 3a. Gonadal and chromosomal characteristics of DSD DSD: disorders of sexual development

Common findings suggesting DSD are male appearance with associated abnormalities of genitalia including severe hypospadias with bifid scrotum, undescended testis/testes with hypospadias, bilateral non-palpable testes, and micropenis with chordee (47,48) or female appearance with associated abnormalities of genitalia including enlarged clitoris, posterior labial fusion, and an inguinal/labial mass (49). An initial assessment, based on the location of the gonads and presence or absence of a uterus, will provide a provisional clinical diagnosis (45). This information combined with karyotype, will provide the basis for more focussed further investigation. Figures 3a and 3b illustrate chromosomal and gonadal characteristics of DSDs. If no gonads are palpable, all options  are possible. Of these, 46,XX DSD (with 2 ovaries) is the most commonly seen, followed by MGD.  The presence of a uterus and absence of palpable gonads in a virilized female primarily suggest a clinical diagnosis of 21-hydroxylase deficiency. If one gonad is palpable, 46,XX DSD and complete gonadal dysgenesis are ruled out because ovaries and streak gonads do not descend. MGD, ovotesticular, and 46,XY DSD remain as diagnostic possibilities. If two gonads are palpable, 46,XY DSD and  ovotesticular DSD are the most likely diagnoses. Symmetrical external genitalia, with or without palpable gonads, and an absent uterus suggest an undervirilized XY male. The presence of a uterus and asymmetric external genitalia and palpable gonad(s) suggest gonadal dysgenesis with Y and ovotesticular DSD. A gonadal biopsy is required to classify the type of gonadal dysgenesis and ovotesticular DSD, to assess gonadal chromosomal mosaicism and to detect the presence of a gonadal tumor. 

Hormone measurements should  be interpreted in relation to specific assay characteristics and also considering  normal values for gestational and chronological age. In some cases serial measurements may be needed. The results of decision making algorithms are available to guide further investigation. These include hCG and ACTH stimulation tests to assess testicular and adrenal steroid biosynthesis. The endocrine evaluation of patients with 46,XY DSDs and sex chromosome DSDs include assessment of testicular function by basal measurement of LH, FSH, inhibin B, T, DHT, AMH, A, and  DHEAS. In patients with T synthesis defects, neonatal and post pubertal diagnosis is made based on  basal steroid levels. The stimulation of T production by hCG is used to pinpoint abnormalities in T biosynthesis and to detect functioning testicular tissue. Testosterone, DHT, A should be measured at baseline and 72 hours after hCG stimulation. The T increment should be at least threefold (56). A failure to respond to hCG in combination with elevated LH/FSH levels and low/undetectable value of AMH is consistent with anorchia or gonadal dysgenesis. Androgen insensitivity should be considered in individuals with a 46,XY karyotype and with normal T biosynthesis. The diagnosis of androgen insensitivity is difficult in absence of a defined androgen receptor mutation. Patients with 5a-reductase deficiency have normal T levels, low or normal DHT levels and a high T/DHT ratio after hCG stimulation test. The diagnosis of 17bHSD deficiency is made when a 10-15-fold elevation is observed in the ratio of A/T. Minimum A/T ratio in a cohort of 24 individuals with a confirmed mutation was 0.7 (57). Inhibin B and AMH are useful markers for the presence of Sertoli cells and their assessment could help in the diagnosis of testis determination disorders. In boys with bilateral cryptorchidism, serum AMH and inhibin B correlate with the presence of testicular tissue and undetectable values are highly suggestive of absence of testicular tissue (58). In XY patients, AMH was found low when the intersex condition was caused by abnormal testicular determination (including complete and partial gonadal dysgenesis) but was normal or elevated in patients with impaired T secretion, whereas serum T was low in both groups. AMH was also elevated during the first year of life and at puberty in intersex states caused by androgen insensitivity. In 46,XX patients with ambiguous genitalia, a serum AMH level  higher than 75 pmol/L is indicative of the presence of testicular tissue and correlates with the mass of functional testicular parenchyma. In conclusion, serum AMH determination is a powerful means  to assess Sertoli cell function in children with intersex , and helps to distinguish between defects of  abnormal testicular determination and of isolated impairment of T secretion or action. 

The diagnosis of 21-hydroxylase deficiency in 46,XX DSDs with two ovaries relies on the detection of elevated 17-OHP levels either as a basal measurement or after a short ACTH stimulation test. High concentration of 11-deoxycortisol and deoxycortisol (DOC) with low levels of plasma renin activity (PRA) will help differentiate  11- from  21-hydroxylase deficiency. 

Supported by good facilities for investigation, clinicians can follow an algorithm that in many cases will lead to an aetiological diagnosis, but with the spectrum of findings and diagnosis, no single evaluation protocol can be recommended in all circumstances 59,60). 

**Figures 4:**
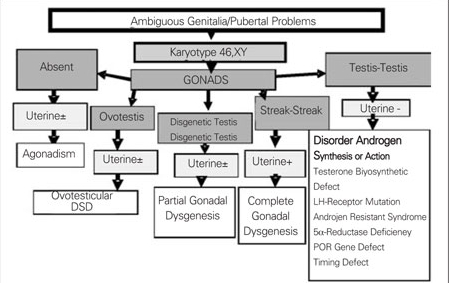
Figure 4. Diagnostic algorithm of 46,XY DSD for new classification DSD: disorders of sexual development

Chromosomal characteristics, gonadal histology and presence or absence uterus are taken into consideration in the classification of DSDs. In our department, we use the diagnostic algorithm prepared in accordance with the new classification for 46,XY DSD and for 46,XX DSD, as given  in Figures 4 and 5. 

**Management** 

The psychological and social implications of gender assignment and those relating to treatment are very important and require a multidisciplinary approach with the inclusion of geneticists, neonatologists, endocrinologists, gynaecologists, psychiatrists, surgeon and social workers in the team. The members of such a team should have a special interest in DSD and  possess sufficient experience with this group of patients. The current intense debate on the management of patients with intersexuality and related conditions focuses on four major issues, namely, aetiological diagnosis, assignment of gender, indications for  and timing of genital surgery, and disclosure of medical information to the patient (61,62,63,64,65,66,67,68, 69,70,71,72,73). Gender identity is a multifactorial process involving both prenatal and postnatal variables. Psychosexual development is influenced by multiple factors such as exposure to androgens, sex chromosome genes, social circumstances and family dynamics. Outcomes can be influenced by timing, dose and type of androgen exposure, receptor availability, and modification by the social environment. Pre- and postnatal hormonal conditions, sex rearing, timing of sex reassignment and corrective surgery appear to be important components for the development of gender-role behavior and gender identity in DSD patients. Karyotype, gonadal function, phenotype, internal genitalia (i.e. presence of uterus), potential for fertility and sexuality, risk of future malignancy, and prenatal brain virilization are some of the many factors which must be taken into account when assessing gender in a child with DSD. Each patient should be evaluated individually by a multidisciplinary approach. Gender assignment should be done after completion of the diagnostic process, including full clinical, genetic, biochemical and psychiatric investigation. The whole procedure should be fully explained to the parents and they should partake in the discussions and decisions. 

The traditional gender assignments for some medical conditions leading to DSDs are as follows: assignment of female gender for 46,XX DSD resulting from fetal androgen exposure, CAIS, and 46,XY complete gonadal dysgenesis; assignment of male gender for 46,XY cloacal exstrophy; assignment of male or female gender for PAIS, 5a-reductase deficiency, ketoreductase deficiency, 46,XY partial gonadal dysgenesis, MGD and ovotesticular DSD. Underlying endocrine disturbances are present in most cases and usually require long-term medication. The current recommendation for a girl with CAH is to rear her as a female and perform a feminizing genitoplasty depending on the degree of masculinization. However, male sex assignment may be mandatory in severely virilized 46,XX CAH patients in whom the diagnosis was not made in the early stage.   Recommendations for sex of rearing especially in infants with genital ambiguity, testicular differentiation disorders and Y chromosome continue to be challenging. Genital masculinization is a poor predictor of the masculinization of the brain (63). Patients with PAIS, 5a-reductase and ketoreductase deficiency, partial gonadal dysgenesis, and MGD may be female or predominantly female at birth and generally are raised as females, but they change their social sex to male at puberty. Sex assignment is more problematic in this group. The decision on sex of rearing in ovotesticular DSD should be based on gonadal and internal ductal formation. 

Surgical techniques of 'feminization' and 'masculinization' and their outcomes have also evolved over time (72,73). Surgeons should have both pediatric training and expertise in DSD surgery. Functional outcome should be taken into consideration rather than a strictly cosmetic appearance. In children assigned a male sex, hypospadias or chordee, if present, requires early surgical correction. Scrotal testis/testes should be monitored and, if needed, biopsied, to rule out malignancy. A streak ovary should be removed. The testis/testes may also need to be removed if there is any risk of malignancy on clinical suspicion or biopsy specimens. T replacement may be required at puberty. The testes in patients with DSD raised as females should be removed to prevent malignancy in pre-puberty or after puberty. Feminizing surgery has three main aims: reducing the size of the enlarged masculinized clitoris, reconstructing the female labia, and increasing the opening and if possible, the  length of the vagina.  Early surgery should only be considered in cases of severe virilization (Prader III-V) and should be carried out in conjunction, when appropriate, with repair of the common urogenital sinus. As orgasmic function and erectile sensation may be disturbed by clitoral surgery, the surgical procedure should be anatomically based to preserve erectile function and the innervation of the clitoris. Consensus conferences recommend not to perform genital surgery between 12 months and adolescence (except for compelling medical indications). Vaginal dilatation should not be recommended before adolescence. 

**3b:**
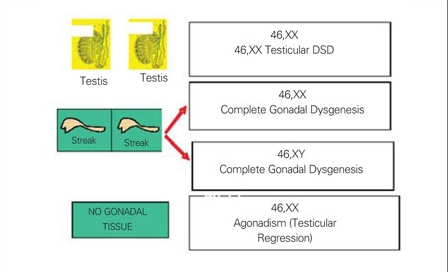
Figure 3b. Gonadal and chromosomal characteristics of DSD DSD: disorders of sexual development

**5:**
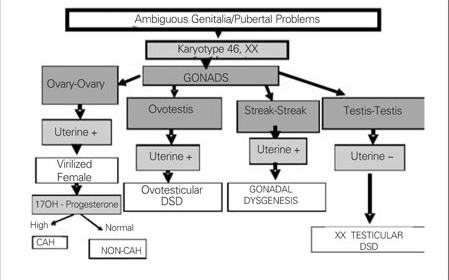
Figure 5. Diagnostic algorithm of 46,XX DSD for new classification DSD: disorders of sexual development, CAH: congenital adrenal hyperplasia

**Table 1:**
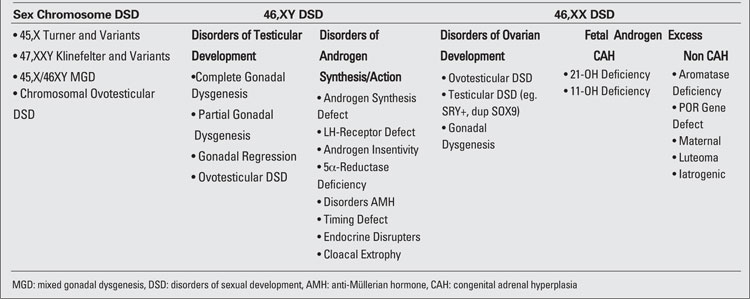
Table 1.  Disorders of sexual development (New DSD nomenclature) (3)
